# Decoding dynamic miRNA:ceRNA interactions unveils therapeutic insights and targets across predominant cancer landscapes

**DOI:** 10.1186/s13040-024-00362-4

**Published:** 2024-04-17

**Authors:** Selcen Ari Yuka, Alper Yilmaz

**Affiliations:** 1 Department of Bioengineering, Yildiz Technical University, Istanbul, 34220 Turkey; 2 Health Biotechnology Joint Research and Application Center of Excellence, Yildiz Technical University, Istanbul, 34220 Turkey

**Keywords:** miRNA, Competing endogenous RNAs, Biological networks, Cancer

## Abstract

Competing endogenous RNAs play key roles in cellular molecular mechanisms through cross-talk in post-transcriptional interactions. Studies on ceRNA cross-talk, which is particularly dependent on the abundance of free transcripts, generally involve large- and small-scale studies involving the integration of transcriptomic data from tissues and correlation analyses. This abundance-dependent nature of ceRNA interactions suggests that tissue- and condition-specific ceRNA dynamics may fluctuate. However, there are no comprehensive studies investigating the ceRNA interactions in normal tissue, ceRNAs that are lost and/or appear in cancerous tissues or their interactions. In this study, we comprehensively analyzed the tumor-specific ceRNA fluctuations observed in the three highest-incidence cancers, LUAD, PRAD, and BRCA, compared to healthy lung, prostate, and breast tissues, respectively. Our observations pertaining to tumor-specific competing endogenous RNA (ceRNA) interactions revealed that, in the cases of lung adenocarcinoma (LUAD), prostate adenocarcinoma (PRAD), and breast invasive carcinoma (BRCA), 3,204, 1,233, and 406 ceRNAs, respectively, engage in post-transcriptional intercommunication within tumor tissues, in contrast to their absence in corresponding healthy samples. We also found that 90 ceRNAs are shared by the three cancer types and that these ceRNAs participate in ceRNA interactions in tumor tissues compared to those in normal tissues. Among the 90 ceRNAs that directly interact with miRNAs, we uncovered a core network of 165 miRNAs and 63 ceRNAs that should be considered in RNA-targeted and RNA-mediated approaches in future studies and could be used in these three aggressive cancer types. More specifically, in this core interaction network, ceRNAs such as GALNT7, KLF9, and DAB2 and miRNAs like miR-106a/b-5p, miR-20a-5p, and miR-519d-3p may have potential as common targets in the three critical cancers. In contrast to conventional methods that construct ceRNA networks using differentially expressed genes compared to normal tissues, our proposed approach identifies ceRNA players by considering their context within the ceRNA:miRNA interactions. Our results have the potential to reveal distinct and common ceRNA interactions in cancer types and to pinpoint critical RNAs, thereby paving the way for RNA-based strategies in the battle against cancer.

**Supplementary Information:**

The online version contains supplementary material available at 10.1186/s13040-024-00362-4.

## Introduction

The most common cancer types in the world are breast, prostate, and lung cancer, according to the latest cancer reports [16]. Therapeutic approaches for cancer types differ according to tissue-specific conditions, the microenvironment, and cellular molecular regulations. Conventional combination therapy approaches such as chemotherapy and radiotherapy can not always overcome the aggressive nature of cancer types. Therefore, authorities from distinct perspectives such as immunotherapy, smart chemotherapeutics, biomolecules, etc., are exerting intense efforts to develop therapeutic approaches [4, 14, 24, 49]. Conversely, the advancement of highly effective cancer therapeutic strategies is intricately linked to a profound comprehension of cancer molecular biology [36]. However, data from emerging molecular techniques have revealed the enrichment and complexity of cancer molecular mechanisms [43].

MicroRNAs (miRNAs) are among the most important regulators of cancer molecular mechanisms. As a crucial class of non-coding RNAs, miRNAs mediate the post-transcriptional regulation of protein-coding mRNAs abundance and cross-talk with other non-coding RNAs, serving as hidden orchestrators for critical biological processes [1, 3]. During this mediating role of miRNAs between both non- and protein-coding RNAs, depending on the abundance of targeted RNA transcripts, the phenomenon of competition for miRNA binding with each other emerges and has been termed competing endogenous RNAs (ceRNAs) [39, 46, 58]. Furthermore, early evidence of ceRNA cross-talk suggested that the competitive behavior of the pseudogene PTENP1 against the tumor suppressor PTEN may be involved in tumorigenesis and could even regulate cancer-associated signaling pathways through competition between protein-coding RNAs [38, 46].

The small-scale miRNA:ceRNA interactions (interactions between a few miRNA:ceRNAs) focused on understanding the dynamics of miRNA:ceRNA interactions have gradually given way to inquiries into the assembly of large-scale miRNA:ceRNA networks and understanding their functions [31]. As a result, miRNA:ceRNA interaction networks have been established by integrated analysis of co-expressed RNAs that are negatively correlated with expression of shared miRNAs, usually based on differential expression analysis, thus elucidating disease- or tissue-specific ceRNA functions and critical players [9, 15, 62]. Several statistical methods have also been suggested for detecting ceRNA interactions based on various correlation methods (i.e., sparse partial correlation (SPC), sum correlation, correlation-based network, and dynamic correlation) [20, 23, 31, 52]. As a result, databases and distinct web interfaces providing ceRNA interactions in cancer tissues have been suggested [19, 48, 50].

In this study, we focused on the fluctuations in miRNA:ceRNA interactions in tumor tissues compared to healthy tissues to provide a comprehensive and deeper perspective on the cancer types with the highest incidence. Using transcriptomic datasets obtained from the TCGA, we first compiled ceRNA interactions in healthy tissues from the breast, prostate, and lung and then in cancer samples of these tissues using sparse partial correlation. We then performed both a comparison of these cancers with healthy tissues and a comprehensive analysis of ceRNA interaction profiles between cancer tissues. In accordance with the complex and ambiguous nature of ceRNA interactions, apart from integrating differentially expressed miRNAs and ceRNAs, or miRNA:ceRNA interaction network measurements, the partial correlation-based workflow was conducted in this study. The results of the study revealed that the miRNA:ceRNA interaction consisting of 63 genes and 165 miRNAs is common to all three cancer tissues and that the GALNT7, KLF9 and DAB2 genes may be involved in critical ceRNA interactions in all three cancer tissues.

## Results

### Tissue-specific ceRNA interactions in healthy tissues

Analysis of miRNA and RNA sequencing data and the miRNA:target interaction matrix revealed that ceRNA interaction dynamics changed in each tissue. For miRNA and RNA transcriptomic data of healthy lung tissue, 46 and 59 tissue samples, respectively, were obtained from TCGA. As a result of the sparse partial correlation performed with these datasets, 393 significant ceRNAs (*p*-value < 0.05) were detected in the lung tissue. Using the ENCORI dataset, the main miRNA:ceRNA interaction network was compiled and contained 444 connections with 87 miRNAs of which these ceRNAs interacted as picked at Table 1. To examine this large-scale network as the subnetworks, betweenness centrality was used and the splitting as sub-networks were performed by considering this measurement. As a result of the clustering, the multiple miRNA:ceRNA criterion was considered in the networks. In other words, subnetworks with large numbers of miRNAs/ceRNAs clustered around a single miRNA/ceRNA or less than 3 nodes were excluded. When subnetworks with multiple miRNA:ceRNA interactions were examined after clustering, 5 miRNA:ceRNA subnetworks emerged in the lung tissue (Fig. S2).

**Table 1:** Overall ceRNA interaction content of healthy and tumor tissues

Tissues	Type	ceRNAs	miRNAs	Connections
Lung	healthy	393	87	444
	tumor	3,270	641	44,605
Prostate	healthy	1,406	637	21,141
	tumor	1,769	639	28,775
Breast	healthy	505	614	6,693
	tumor	454	618	8,878

Interestingly, for prostate tissue with an equal number of miRNA and RNA sequencing data (i.e. 52 samples), a much larger miRNA:ceRNA interaction main network was identified. After clustering, 13 subnetworks were generated and a significant proportion of the crucial miRNAs and ceRNAs in healthy tissue were found to participate in the main subnetwork. While 8 networks obtained from healthy prostate tissue included limited interactions between a few miRNAs and ceRNAs, 4 miRNA:ceRNA interactions were found to constitute moderate subnetworks (Fig. S3). In the healthy breast, where the largest amount of transcriptomic data was acquired (113 RNA and 104 miRNA samples), sparse partial correlation analysis showed that, unlike other tissues, 505 ceRNAs were interacted by a higher number miRNAs (614) than ceRNAs and involved 6,693 interactions. Clustering resulted in one large-scale containing 67 miRNAs and 85 ceRNAs, and 5 other subnetworks in breast tissue (Fig. S4).

### ceRNA interactions in tumor tissues

Sparse partial correlation on transcriptomic data and interaction matrices revealed completely dissimilar ceRNA interactions in lung (LUAD), prostate (PRAD) and breast (BRCA) cancers compared to those in normal tissues. In lung cancer, which has a significantly greater transcriptomic data content (i.e., 519 miRNA and 539 RNA expression samples) than in normal tissue samples, the analysis showed that 3,270 ceRNAs participated in 44,605 interactions across 641 miRNAs, and 24 subnetworks were obtained from this large-scale interaction by betweenness centrality-dependent clustering (Table 1). Furthermore, in the context of lung adenocarcinoma, it’s worth noting that miRNA:ceRNA interactions predominantly occur as local interactions by subnetworks involving relatively fewer miRNA and ceRNA components than in normal tissue.

On the other hand, there were 498 and 501 miRNA and RNA transcriptomic samples, respectively, for PRAD tissue, and analysis of these data revealed that the large-scale network of PRAD tissue involved 28,775 interactions comprising 639 miRNAs and 1,769 ceRNAs (Table 1). As in healthy tissue, among the subnetworks derived from PRAD samples (number of subnetworks, 17), the large-scale subnetwork of 1,851 connections between 127 miRNAs and 237 ceRNAs prevailed and was followed by 5 small-scale subnetworks. The most fascinating findings were obtained from BRCA tissue. RNA and miRNA expression data from 1,111 and 1,096 samples, respectively, were obtained in BRCA tissue, and 8,878 miRNA:ceRNA interactions between 618 miRNAs and 454 ceRNA nodes were revealed, as shown in Table 1. Centrality-dependent clustering revealed that a significant proportion of cross-talking miRNAs and ceRNAs participate in a major large-scale subnetwork, and only two networks (which contain only a few interactions) may participate in miRNA:ceRNA interactions (total subnetwork = 11).

### Lung adenocarcinoma vs healthy lung tissues

Unlike the ceRNA interactions found in normal tissues, 3,204 LUAD-specific ceRNAs were identified. In addition to the common 66 ceRNAs involved in ceRNA interactions in normal tissues, 327 genes were not involved in ceRNA activity in tumor tissues. DEG analysis of these ceRNAs revealed 113 downregulated genes and 248 upregulated genes. Among the fluctuating ceRNAs, those that were analyzed by DEA (padj < 0.05) were found to be significantly associated with many cancer-related hallmarks and KEGG pathways. Particularly noteworthy was the enrichment in the KEGG cell cycle (*p*-value: 0.0037) and purine metabolism (*p*-value: 0.0006). Consistent with this, the enrichment in G2M checkpoint, E2F transcription factors, and MYC targets suggest that clustered ceRNA interactions are involved in the function of cancer cell proliferation (Supplementary Fig. S6).

**Fig. 1:**
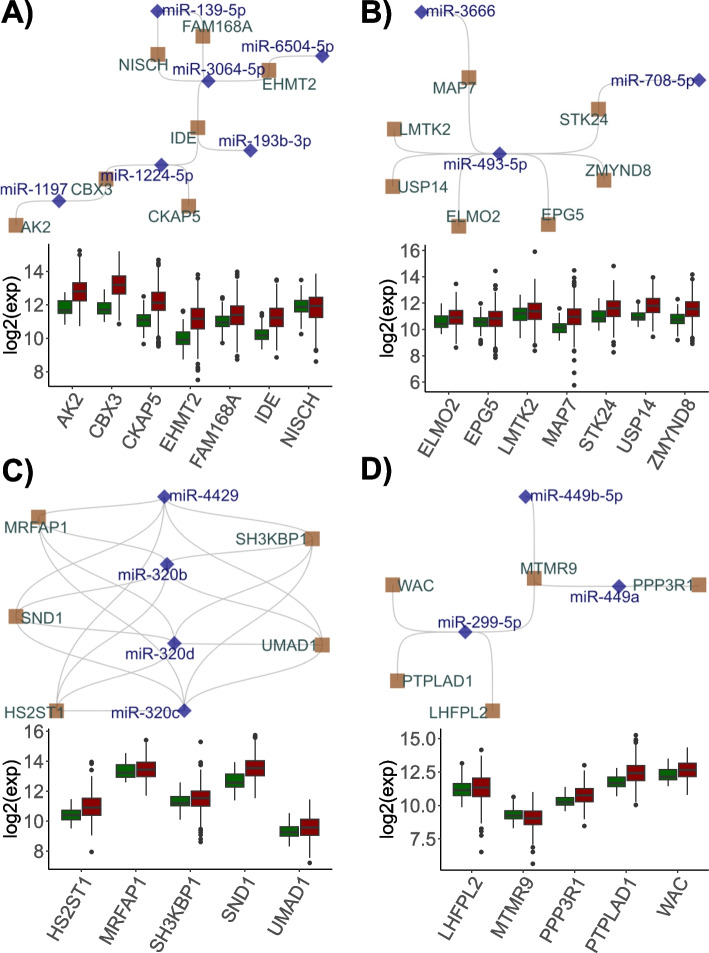
LUAD-specific miRNA:ceRNA networks, miRNAs and ceRNAs are shown with blue diamonds and ceRNA brown squares, respectively. The log2 transformed expression values (y-axis) of ceRNAs (x-axis) in tumors (red) and healthy (green) tissues are shown in box plots

The miRNA:ceRNA networks consisting of miRNAs interacting with these ceRNA nodes were analyzed. A total of 3,204 ceRNAs were observed to participate in an interaction network comprising 42,829 connections with 641 miRNAs. To analyze this multi-layered cross-talk as submodules, clustering was implemented according to betweenness centrality measurement. As a result, most miRNAs and genes involved in LUAD-specific miRNA:ceRNA interactions were shown to participate in a large-scale subnetwork with 167 miRNAs and 363 ceRNAs, and 7 miRNA:ceRNA interaction subnetworks were also distinguished. The most outstanding of the networks are shown in Fig. 1A-D and the other three are depicted in Fig. S5 in the Supplementary file.

Among these 7 subnetworks, a subnetwork of 6 miRNAs (miR-1197, miR-1224-5p, miR-3064-5p, miR-139-5p, miR-6504-5p, and miR-193b-5p) and 7 ceRNAs (CBX3, EHMT2, AK2, IDE, CKAP5, EHMT2, NISCH, and FAM168A). The functions of Chromobox protein homolog 3 (CBX3), which is known to be especially critical in epigenetic mechanisms, have been explored in various cancer tissues and recent studies have emphasized that CBX3 has prognostic significance in non-small cell lung cancer and is significantly associated with poor overall survival in patient with lung cancer [30, 56]. Among the limited number of studies, Yan et al. constructed weighted gene correlation networks with mRNAs and lncRNAs negatively co-expressed with shared miRNAs in the TCGA LUAD dataset [60]. As a result, CBX3 was shown to display a prominent interaction module with the protein-coding gene P4HA1 and lncRNAs, namely AC032632.6, AC026356.1, and LINC02535, mediated by miR-30b-5p and miR-30d-5p.

Additionally, EMHT2 interacted with CBX3 through the CBX3/miR-1224-5p/IDE/miR-3064-5p/EHMT2 axis. Similarly, compared with that in normal tissue, the expression of miR-1224 in this axis is generally downregulated in lung tumor tissue [33], although the tumorigenic, angiogenic, and carcinoma progression functions of miR-3064 in different cancer types have been explained, its functions in lung cancer have not been clarified [55, 63]. Other ceRNAs involved in this interaction, such as Cytoskeleton-associated protein 5 (CKAP5) and Adenylate kinase 2 (AK2), have been reported to regulate characteristic cancer biological processes, particularly in pathways such as microtubule stabilization or TGF-$\beta$/Smad signaling [5, 7]. While there are a limited number of reports in the literature addressing the ceRNA regulatory function regulation of these genes in cancer tissue and their interactions with several non-coding genes, our analyses strikingly revealed that miRNAs may regulate possible tumor-associated cross-talk between these genes, which may act as ceRNAs reported in diverse studies.

### Postate adenocarcinoma vs healthy prostate tissues

In prostate cancer, the number of genes observed in PRAD-specific ceRNA interactions that were not found in normal tissues was 1,233. However, the sparse partial correlation analysis of prostate tissue samples revealed a high number of common ceRNAs in tumor and healthy tissues (n=536), while 870 ceRNAs did not exhibit competitive behavior in tumor tissue. DEG analysis with these PRAD-specific ceRNAs strikingly revealed differences in the expression of only 81 genes, 41 upregulated and 40 downregulated genes. Functional annotation revealed that these genes were significantly enriched in the tumor-associated KEGG arginine and proline metabolism (*p*-value: 0.015) and hallmark epithelial to mesenchymal transition (*p*-value: 0.036) (data not shown).

The network of 1,233 ceRNAs with miRNA interactions contained 17,299 connections. Clustering from this large-scale network according to betweenness centrality yielded 11 small-scale miRNA:ceRNA interactions in addition to a large-scale subnetwork between 335 ceRNAs and 217 miRNAs. In contrast to LUAD tissue, subnetworks in PRAD tissue exhibited small-diameter miRNA:ceRNA interactions, except for one dedicated interaction involving 5 miRNAs and 7 ceRNAs. Therefore, these small-diameter miRNA:ceRNA interactions were not subjected to subsequent analyses.

**Fig. 2:**
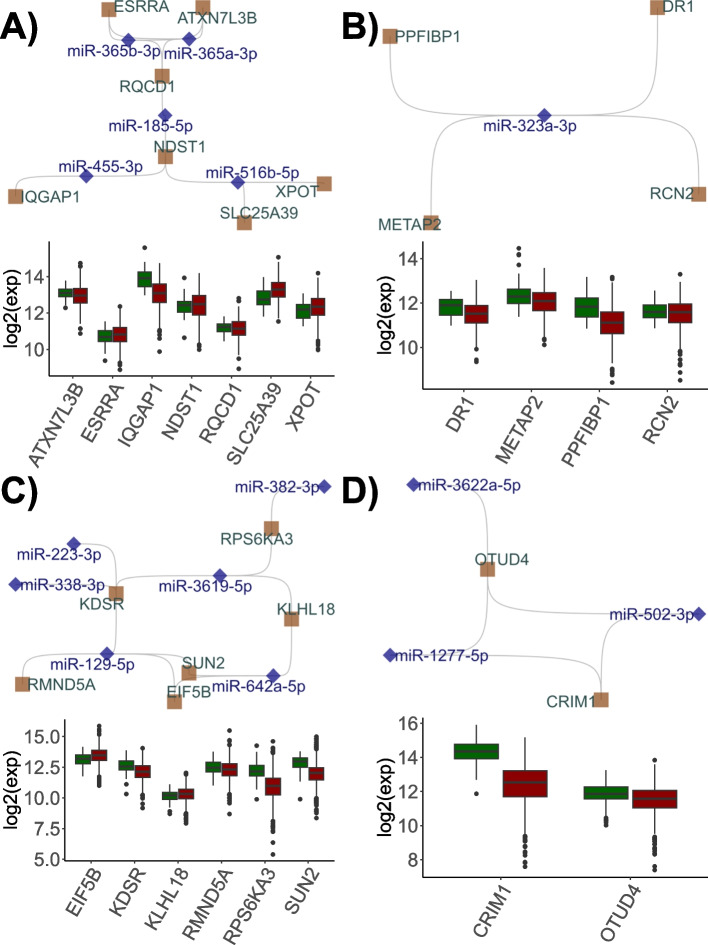
PRAD- (**A**, **B**) and BRCA- (**C**, **D**) specific ceRNA subnetworks. miRNAs and ceRNAs are shown with blue diamonds and ceRNA brown squares, respectively. The log2-transformed expression values (y-axis) of ceRNAs (x-axis) in tumors (red) and healthy (green) tissues are shown in box plots

Among the PRAD-specific miRNA:ceRNA subnetworks, a subnetwork involving interactions between 5 miRNAs (miR-455-5p, miR-516b-5p, miR-185-5p, and miR-365a/b-3p) and 7 ceRNAs (SLC25A39, XPOT, IQGAP1, NDST1, RQCD1, ESRRA, and ATXN7L3B) was found (Fig. 2A and B). Although this interaction shows ceRNA potential in sparse partial correlation analysis, it is distinct from the network observed in LUAD in terms of its expression change dynamics. In this critical miRNA:ceRNA interaction, the changes in the expression of ceRNAs exhibit a discordant profile. There are various reports that genes involved in this interaction such as SLC25A39, NDST1, and RQCD1 regulate energy metabolism, chemoresistance, and the Akt signaling pathway, respectively, in cancer tissues [17, 18, 64]. However, there is a significant gap in studies investigating the comprehensive function of ceRNAs. In this respect, our findings suggest that this cross-talk should be evaluated in the regulation of these genes or miRNAs in PRAD.

### Breast cancer vs healthy breast tissues

The fluctuating ceRNA interactions in breast cancer have completely distinct characteristics from those in other tissues. A total of 457 ceRNAs, a significant portion of the ceRNA interactions obtained as a result of ceRNA sparse partial correlation in healthy tissue, were not observed in the BRCA ceRNA interactions. Forty-eight common and 406 BRCA-specific ceRNAs were found between tumor and healthy tissues. When 406 BRCA-specific ceRNAs were subjected to DEA, only 44 genes exhibited significant differential expression. Among the DEGs, 13 were down- and 31 up-regulated (|log(FC)| > 1 and padj < 0.05). The significant enrichment in the hallmark G2M checkpoint (*p*-value: 0.007) and mitotic spindle (*p*-value: 0.004), and in the KEGG neuroactive ligand-receptor interaction (*p*-value: 0.03) were noteworthy (Fig. S7).

However, a network of 406 BRCA-specific ceRNAs with many miRNAs (n=617) prevailed. Clustering by betweenness centrality revealed several small diameter miRNA:ceRNA interactions in addition to a large-scale subnetwork in breast cancer as in the LUAD and PRAD tissues. This large-scale miRNA:ceRNA subnetwork contained 167 miRNAs and 129 ceRNAs. In addition, two subnetworks emerged in BRCA, containing 6 miRNAs:6 ceRNAs and 3 miRNAs:2 ceRNAs (Fig. 2C and D).

One of the first interactions that emerged in breast cancer tissue compared to normal tissue was the interaction between RMND5A, EIF5B, SUN2, KLHL18, KDSR, RPSK6KA3 ceRNAs and the miR-338-5p, miR-223-3p, miR-129-5p, miR-642a-5p, miR-3619-5p, and miR-382-5p miRNAs. Interestingly, nodes in this small-scale miRNA:ceRNA interaction have tissue-specific expression and/or function. For example, miR-21-mediated regulation of RMND5A expression might be associated with survival in patients with breast cancer [6]. Conversely, miR-590-mediated decreases in the expression of this gene may result in the inhibition of metastatic pathways in pancreatic adenocarcinoma [10]. Similarly, the SUN2 gene, which has distinct tumor-specific functions, has also been implicated in these interactions. It has been emphasized that decreased expression of the SUN2 gene in breast cancer tissues may be critical for cancer prognosis [8]. In this interaction, KLHL18 has a tumor suppressor function, while KDSR is active through sphingolipid metabolism and its inhibition causes endoplasmic reticulum dysfunction specific to breast cancer cells [13, 21]. In general, the number of ceRNAs involved in this interaction tends to increase, while the expression of EIF5B, which is associated with poor prognosis, is slightly increased in tumor tissue [45].

### Comparison of tumor-specific ceRNA interactions

**Fig. 3:**
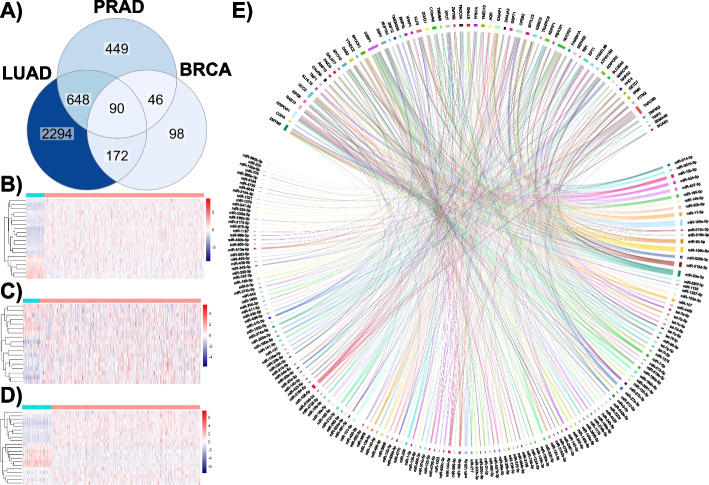
Intersected tumor-specific ceRNAs across LUAD, PRAD, and BRCA. Venn diagram (**A**) of tumor-specific ceRNAs in three cancer types. Comparison of the expression profiles of common (n=90) ceRNAs in LUAD (**B**), PRAD (**C**), and BRCA (**D**) tissues from healthy individuals and tumor tissues. Blue and pink indicate healthy and tumor samples, respectively. Chord diagram (**E**) of core common miRNA:ceRNA interactions in LUAD, PRAD, and BRCA

We evaluated miRNA:ceRNA interactions in tumors compared to healthy tissues. For each tissue type (i.e., lung, prostate, and breast), only the ceRNAs involved in significant ceRNA interactions in tumor tissues were the subject of the analysis. By overlaying the tumor-specific ceRNAs in all three cancer types, 90 ceRNAs were found to participate in interactions in all cancer types. Functional annotation of these 90 ceRNAs via DAVID revealed that they are significantly involved in diverse biological processes and molecular functions [41] (Fig. S8). In addition, when the expression profiles of these genes in tumor and healthy tissue samples were examined, it was found that while a significant expression pattern was observed in healthy tissues in general, there was an insignificant and fluctuating expression pattern in tumor tissues. However, it cannot be argued that there was a concordance in the changes in the expression of 90 genes involved in ceRNA interactions across three tissues (Fig. 3B-D). This may indicate that it is possible to pinpoint critical genes that cannot be detected by DEG analysis.

Interestingly, in all three tumor types, a high number of miRNAs (n=492) were observed to participate in miRNA interactions with the common 90 ceRNAs that occur in cancer tissue unlike in normal tissues (Fig. 3A). When this subnetwork was overlaid with the DEG analysis results of 90 genes, a co-regulation fashion was observed especially in particular genes. The most significant change was observed in KIF11 with log2FCs of 2.58, 0.90, and 2.39 in LUAD, PRAD, and BRCA, respectively as shown in Table 2. On the other hand, no significant downregulated ceRNA pattern was observed in any of the tissues in this subnetwork. The main reason for this difference is that the downregulation was slight, especially in PRAD tissue (Table 2). All details of these common 90 ceRNAs are given in the Supplementary data.

**Table 2:** Differential gene expression profile of common ceRNAs in the subnetwork. The values in LUAD, PRAD, and BRCA variables refer to the log2(fold change) of the given ceRNAs

ceRNA	LUAD	PRAD	BRCA
KIF11	2.58	0.90	2.39
FANCA	1.90	0.64	1.79
GALNT7	1.60	0.95	1.15
ALG3	1.08	0.52	1.12
LEPR	-1.95	-0,80	-2.97
KLF9	-1.76	-0.39	-1.76
DAB2	-1.16	0.30	-1.03

Additionally, a core subnetwork consisting of 165 miRNAs and 63 ceRNAs was revealed, when isolated nodes in this network were excluded (Fig. 3E). In this core network, miRNAs with the highest centrality degrees among 165 miRNAs participating in cross-talk of 63 ceRNAs were miR-106a-5p, miR-20a-5p, miR-106b-5p, miR-519d-3p, and miR-17-5p (Supplementary data). Some of the ceRNAs given in Table 2 (LEPR, ALG3, KIF11, and FANCA) were found to be isolated in miRNA interactions formed by 90 common ceRNAs. On the other hand, our analysis showed that the GALNT7, KLF9, and DAB2 genes may be particularly critical as ceRNAs in three cancer tissues. Notably, there were no notable changes in the expression of ceRNAs with a high degree of centrality (Supplementary data). It would not be argued that these findings are entirely contrary to the nature of the ceRNA hypothesis. This is because the ceRNA hypothesis is principally explained by the fact that a change in the expression of one ceRNA in the network through shared miRNAs causes the expression of other nodes to be regulated in the same fashion [39, 46]. In this case, as the number of interactions (connections) between miRNAs and ceRNAs increases, the amount of transcript change required for a significant effect greatly increases due to the growing number of shared ceRNAs and miRNAs. Therefore, in ceRNA interactions that involve a tremendous amount of cross-talk, a significant level of perturbation (e.g. when the expression of a gene increases too much) can only yield minor changes at the transcriptomic level. However, the importance of these small changes in biological processes is extremely uncertain.

## Methods

The study relies on the collection of transcriptomic data and miRNA:target interactions, the sparse partial correlation using these files. This comparative and comprehensive analysis of miRNA:ceRNA interactions in tissues (lung, prostate, and breast) and conditions (tumor vs healthy) revealed the fluctuating miRNA:ceRNA interactions. The general workflow of the study is depicted in Fig. S1.

### Data acquisition

The miRNA isoform and RNA sequencing data (as counts) for normal and tumor samples of breast, prostate, and lung tissues were obtained from the TCGA database using the TCGAbiolinks and SummarizedExperiment R packages [12, 34, 35]. All the data cleaning and manipulation stages were performed using standard R Tidyverse functions [54].

The miRNA:target pairs were obtained from the ENCORI (The Encyclopedia of RNA Interactomes) database [26]. In the ENCORI database, miRNA targets were grouped into mRNA, lncRNA (lincRNA, processed_transcript, sense_intronic, 3prime_overlapping_ncRNA, antisense, sense_overlapping, bidirectional_promoter_lncRNA), circRNA, sncRNA, and pseudogene. Separate datasets were processed and combined.

### Sparse partial correlation for healthy and tumor samples for three distinct tissues

To identify the tissue-specific miRNA:ceRNA network in healthy and tumor tissues, the SPONGE package based on the sparse partial correlation method was implemented [31]. The log2-transformed miRNA and RNA sequencing count data from six subjects and the miRNA:target matrix were used as input files. As the workflow demands a considerable amount of memory, the SPONGE analysis was performed in a high-performance computing (HPC) environment. The significance threshold was picked as *p*-value < 0.05, and as a result, ceRNA:ceRNA pairs were found for all subjects.

### Construction of tissue- and condition-specific networks

We collected all miRNA:target interactions for potential ceRNA:ceRNA pairs with partial correlations from the ENCORI database. These miRNA:ceRNA datasets were transformed into graph objects using the tidygraph package [37]. Then, we applied network clustering based on edge betweenness, a widely used centrality measure in biological networks [47]. This allowed us to detect the evolving ceRNA regulators within integrated tissue miRNA:ceRNA interaction networks, both as a whole and in subnetworks.

### Differential gene expression analysis

To analyze the relevance of the fluctuation in ceRNA interactions to the change in the expression of ceRNA genes, differential gene expression analysis was performed on the transcriptomic data of normal and tumor samples obtained from the TCGA for three tissues. The DESeq2 package was used for the analyses and the comparisons were based on common or discrete genes according to tissue type or condition (i.e., healthy vs tumor) [32].

### Functional annotation

The statistics from the DEG analysis of specific ceRNA genes in tumor tissues were used as input in the fgsea package and gene set enrichment analysis was performed [25]. Functional annotation analysis of common ceRNAs (*n*=90) obtained from the intersection of ceRNAs detected in tumor tissues apart from normal tissues was performed using DAVID [41].

## Discussion

Following the growing consensus that competitive endogenous RNAs are critical and complex cross-talks involved in the regulation of cellular functions beyond hypothesis, a large number of studies have been conducted to computationally identify the ceRNA:miRNA axes involved in abnormal functions through these mechanisms or to modulate these axes with specific mimics. The potential of the ceRNA interactome for therapeutic purposes has been revealed, particularly in many disease types such as metabolic, cardiovascular disorders, and cancer [44, 66]. In particular, large- and small-scale ceRNA:miRNA interactions revealed from transcriptomics and miRNA:target interaction data have enabled significant progress in cancer treatment. Many of these studies address large- or small-scale interactions in cancer. However, since the ceRNA interactions are characterized by competitive behavior depending on RNA abundance, fluctuations in ceRNA interactions between healthy and tumor tissues are naturally anticipated. In this case, the analysis and discovery of ceRNA interactions in healthy tissues but not in cancerous tissues may reveal novel insights for developing RNA-based cancer therapeutics.

In this study, we analyzed ceRNA-miRNA interactions in healthy and tumor tissues of the cancer types with the highest incidence in the world and revealed common or tumor-specific biased ceRNA interaction dynamics in these cancer types compared with healthy tissues. For this purpose, we compiled the main miRNA:ceRNA interactions in healthy and cancer tissue datasets of lung, prostate, and breast from the TCGA database using sparse partial correlation. Subsequently, ceRNA interactions that are common or specific to tumor tissues were comprehensively deciphered (Fig. S1).

Our analyses revealed that ceRNA candidates from healthy and tumor samples of each tissue type were different, regardless of the number of samples. For the lung, prostate, and breast, the numbers of common ceRNAs between healthy and tumor tissues were 66, 536, and 48, respectively. However, for LUAD, PRAD, and BRCA, the numbers of ceRNAs that distinguished from normal tissues were 3,204, 1,233, and 406, respectively. The most notable fluctuation among cancer types was the ceRNAs that arose in LUAD tissue. We found that almost 7 times more significant ceRNAs participated in LUAD tissue, which contained only 66 common ceRNAs with healthy tissue, according to sparse partial analysis, as shown in Table 1. On the other hand, although a slight increase in the number of ceRNAs was also observed in PRAD tissue, these cells shared a significant number of common ceRNAs (536) with healthy tissue. Interestingly, an approximately 12% lower number of significant ceRNAs was observed in BRCA tissue than in breast tissue. However, the interactions of decreased ceRNAs through miRNAs increased by almost 30% (Table 1).

When the miRNA interactions of these ceRNAs in cancer tissues were compiled and networks were constructed, the unique large-scale miRNA:ceRNA interaction of each tissue resulted in varying numbers of miRNA:ceRNA clusters. DEG analysis for tumor-specific ceRNAs showed that a significant proportion of these tumor-specific ceRNAs had no significant change in expression. In this regard, our analysis is distinct from conventional methods involving the integration of ceRNAs and miRNAs and allows for the flexible perspective to capture missing interactions that may arise from spontaneous RNA-seq data. Notably, gene set enrichment analyses based on statistics of tumor-specific ceRNAs have been limited by the small number of DEGs in tumor-specific ceRNAs. As a consequence, gene set enrichment analyses of LUAD- and BRCA-specific ceRNAs have yielded significant results in several signaling pathways.

The most critical finding of our study was the 90 ceRNAs shared by all tumor types from the unique ceRNAs. When the expression of these ceRNAs was compared between tumor and healthy tissues, no apparent pattern was observed in tumor tissues, while the expression of ceRNAs was concordant with that in normal tissues. These critical findings suggest that the 90 ceRNAs in the three tumor tissues are somehow disrupted and may be related to cancer (Fig. 3B-D). The functional annotation of ceRNAs common to all three tumor tissues showed that ceRNA regulatory roles may be predominant, especially in metabolism (adipocytokine signaling) or AMPK signaling pathways (Fig. S8). Therefore, these 90 ceRNAs and their interacting miRNAs were analyzed, and a many-to-many miRNA:ceRNA network (165 miRNAs and 63 ceRNAs), which is critical for all three cancers, was revealed (Fig. 3E). In this core network, the most crucial miRNAs according to centrality degree measurement were miR-106a-5p, miR-20a-5p, miR-106b-5p, miR-519d-3p, and miR-17-5p. Previous work was consistent with our findings and reports significant roles of these miRNA in cancer [11, 42]. On the other hand, functional annotation of ceRNAs common to all three tumor tissues showed that ceRNA regulatory roles may be predominant, especially in metabolism (adipocytokine signaling) or AMPK signaling pathways (Fig. S8). Considering the findings in the literature in parallel to our large-scale ceRNA analysis, comprehensive analysis of ceRNA fluctuations revealed remarkable results for the integration of the small pieces of the main puzzle in the literature. In future studies, new therapeutic prospects may be offered by artificial ceRNAs (such as circRNA or lncRNA mimics) that can collectively target and regulate this extensive ceRNA cluster.

On the other hand, among the tumor-specific 63 ceRNAs, GALNT7, KLF9, and DAB2, which are common to all three tumor tissues and were significantly expressed in at least two tissues (Table 2), were found in this core miRNA:ceRNA cluster. For example, up-regulation of the GALNT7 gene has been associated with tumor progression in prostate, cervical, and laryngeal cancers [27, 40], but has been the subject of only a limited number of studies in terms of ceRNA interactions [28]. KLF9, the increased expression of which has been reported to have an inhibitory effect on cancer, has been used for therapeutic purposes in various cancer types. Axes with few nodes have been shown to exist, and these axes can be used therapeutically against cell proliferation, invasion, and migration [22, 61, 65]. In addition, KLF9 has been reported to participate in coregulatory ceRNA interactions in bladder urothelial carcinomas [29]. Finally, although the anticancer effect of DAB2, which is common in all three cancer tissues, was first reported in ovarian cancer, DAB2 is regulated by non-coding RNAs in distinct cancer types [51, 59].

Considering the findings in the literature in parallel to our large-scale ceRNA analysis, comprehensive analysis of ceRNA fluctuations revealed remarkable results for the integration of the small pieces of the main puzzle in the literature. Here we report a core network resulting from the condition-specific differences in ceRNA interactions, by using a method that identifies ceRNA interactions that are sensitive to the expression of miRNA targets, shared miRNAs, and the expression of these miRNAs, but sample-specific aspects should be considered. ceRNA interactions can be observed in the conditions of specific stoichiometric thresholds arising from the abundance of miRNAs and their targets, and biological function can only be achieved under those conditions [2]. Therefore, the core network can also be more functional biologically by narrowing it according to the ratios of ceRNA and shared miRNA expressions of relevant sample. Furthermore, various molecular and epigenetic mechanisms, spanning from genomic structural alterations to gene regulatory networks, are implicated in the regulation of protein-coding genes by non-coding RNAs. For example, TF-mRNA-miRNA driven feed-forward loops or copy-number alterations (CNAs) in specific chromosome regions of ceRNAs have been reported to be associated with critical biological processes such as immunity and metastasis hallmarks, respectively [53, 57]. In our study, we reported a shared core miRNA:ceRNA network for 3 critical cancer types compiled according to the sparse partial correlation by using the expression of miRNAs and their targets in distinct tissues and conditions. However, deeper tissue-specific investigation of these interactions by considering TFs or structural alterations may lead to a more targeted focus on miRNA:ceRNA interactions, so new therapeutic prospects may be offered by artificial ceRNAs (such as circRNA or lncRNA mimics) that can collectively target and regulate this ceRNA clusters.

## Conclusion

Our results demonstrate a comprehensive analysis of ceRNA interactions that differ between cancer and healthy tissues, in addition to the miRNA:ceRNA interactions that have been found at large- or small- scales. Conventional ceRNA interactions are typically focused on diseased tissue and are often based on changes in the expression of ceRNAs. However, the perspective of this study provides a deep understanding of the shift of cancer relative to default (i.e. healthy) ceRNA interactions. This integrated study of the most critical cancers has also uncovered the common and distinct modules of ceRNA dynamics that are changed in cancer types. Our results could lead to the development of novel RNA therapeutics that may exhibit common or tissue-specific efficacy in these three cancer types.

### Supplementary Information

**Supplementary Material 1.**

## Data Availability

No datasets were generated or analysed during the current study.
