# Supplementary material for: Decoding dynamic miRNA:ceRNA interactions unveils therapeutic insights and targets across predominant cancer landscapes
Source: BioData Min. 2024 Apr 17;17:11. doi: 10.1186/s13040-024-00362-4 (PMC11022475; doi:10.1186/s13040-024-00362-4)
Supplement: Supplementary file 1 — Supplementary Material 1. [file 13040_2024_362_MOESM1_ESM.zip › Supplementary_material.pdf]

Supplementary Information

Selcen Ari Yuka, Alper Yilmaz

S1. Overview

In this study, RNA and miRNA sequencing data of normal and tumor tissues were subjected to sparse partial correlation analysis via miRNA:target database obtained from ENCORI database. Thus, ceRNA interaction candidates of both healthy and tumor tissues of lung, prostate and breast tissues were found. Hence, apart from healthy tissues, ceRNAs that emerge only in tumor tissues are considered as cancer-specific ceRNAs (step I in Figure S1).

Following the profiling of cancer-specific changed ceRNAs, common and distinct ceRNAs were obtained in the three cancer types and their interactions with miRNAs, their sub-networks and expression changes were comprehensively analyzed (step II in Figure S1).

S2. miRNA:ceRNA interaction subnetworks in normal tissues

We found 46 subnetworks when clustering by considering networks that contain at least 3 nodes in the lung tissue according to the betweenness centrality measure. However, the 5 most prominent networks are shown in Figure S2.

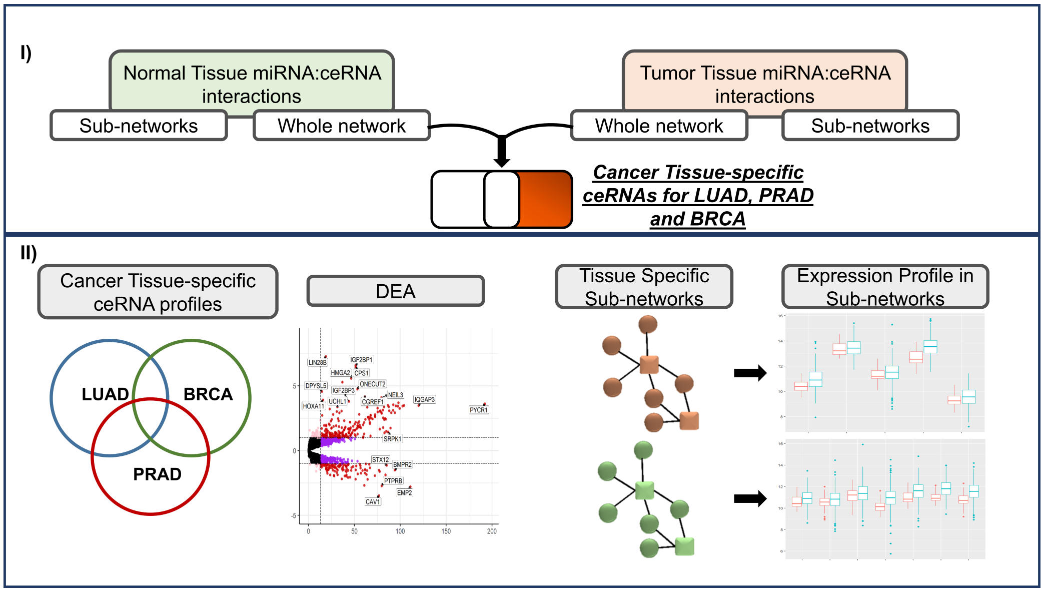

Figure S1: General workflow to decipher shifting ceRNA dynamics in three cancer types

Clustering based on betweenness centrality yielded 13 subnetworks of total consisting of 12 small-scale and a single extensive subnetwork comprising 5,421 connections involving 300 miRNAs and 442 ceRNAs. (Figure S3 large-scale subnetwork was not given here.)

Clustering the main miRNA:ceRNA network into subnetworks based on betweenness centrality produced several distinct subnetworks for BRCA. The most noteworthy among these is subnetwork 1, characterized by 382 interactions involving 67 miRNAs and 85 ceRNAs (Figure S4A). Furthermore, specific interaction patterns were observed between a limited number of miRNA and ceRNA nodes in breast tissue (as indicated in Figure S4 B-E).

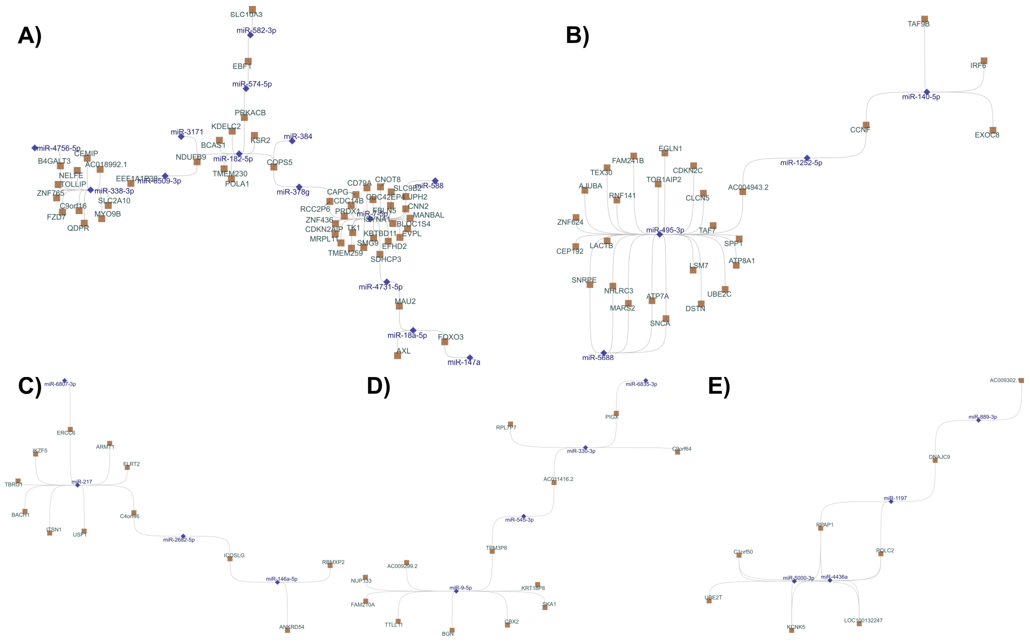

Figure S2: Crucial miRNA:ceRNA subnetworks in lung

S3. Tumor-specific several ceRNA networks and pathway enrichment results

Among the LUAD-specific ceRNA subnetworks, 7 clusters emerged in particular. While 4 of these clusters are shown in the main text, the other 3 are included in Figure S5. It is also noteworthy that LUAD-specific ceRNAs whose differentially expressed were enriched in many cancer-related pathways (Figure S6).

On the other hand, while a significant proportion of BRCA-specific ceRNAs cluster into the large-scale miRNA:ceRNA subnetwork, gene set enrichment analysis of 44 differentially expressed genes resulted in significant enrichment in several hallmarks and KEGG pathways (Figure S7).

S4. Functional annotation of common tumor-specific ceRNAs

Functional annotation of 90 ceRNAs obtained by intersecting cancer-specific ceRNAs from three tissue types was performed using the DAVID database, resulting in Figure S8.

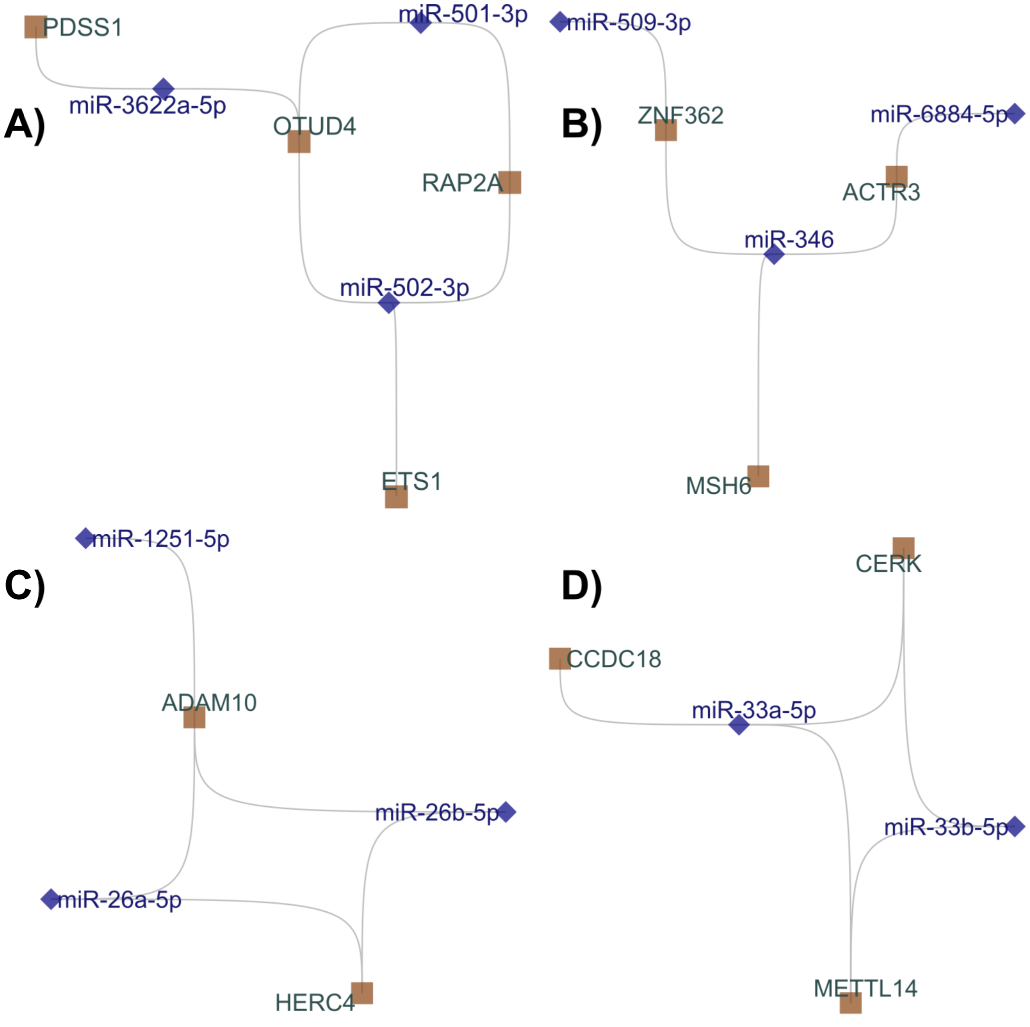

Figure S3: Crucial miRNA:ceRNA subnetworks in prostate

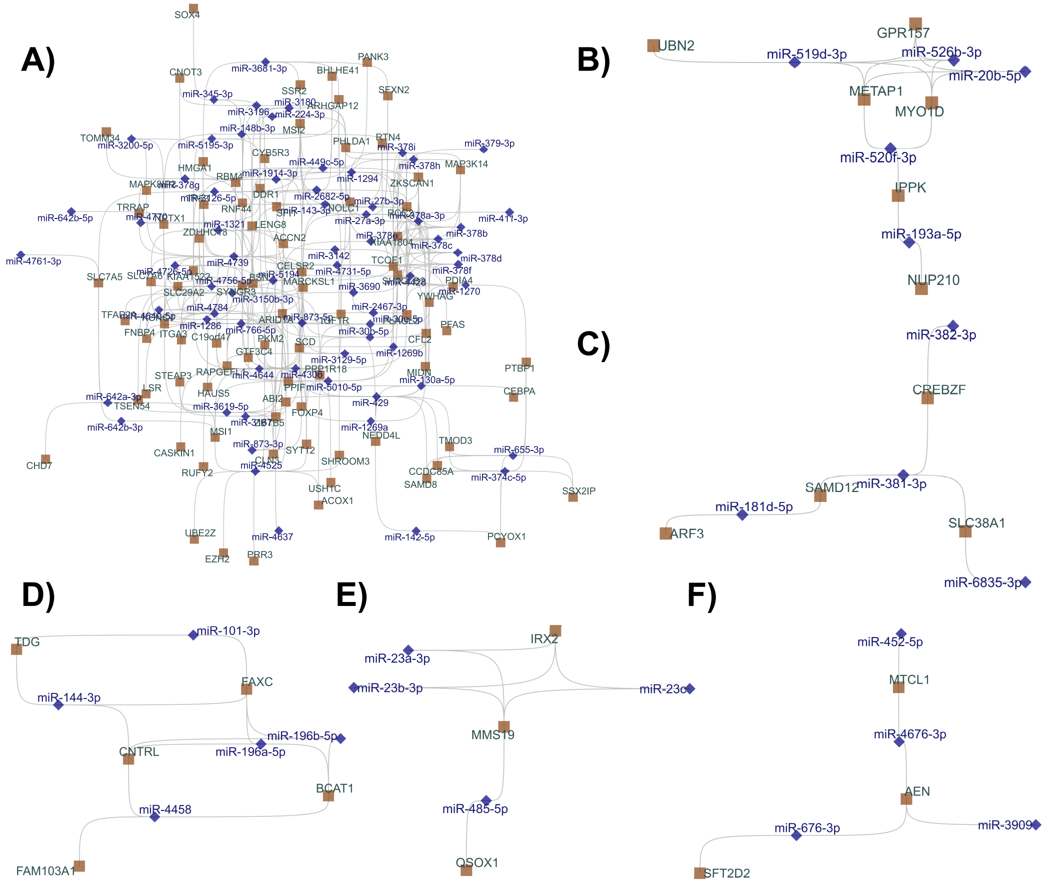

Figure S4: Crucial miRNA:ceRNA subnetworks in breast

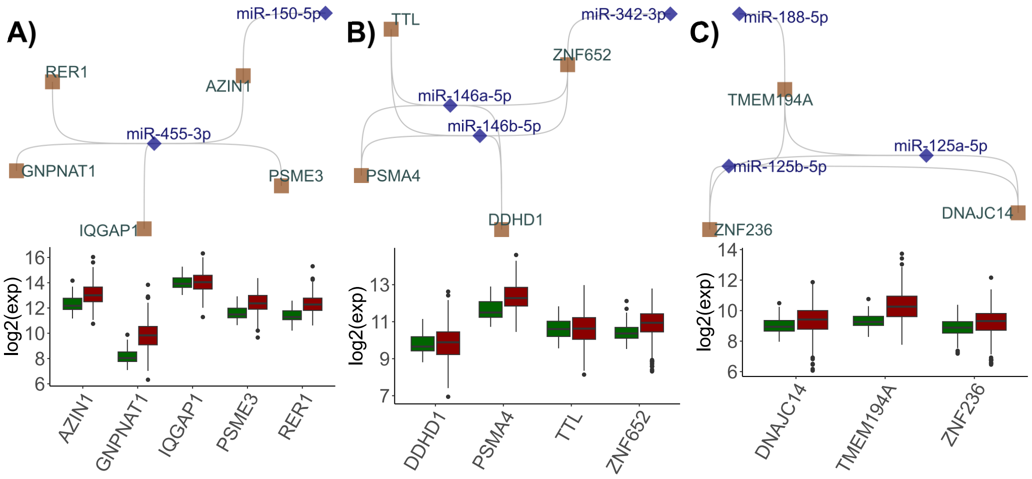

Figure S5: The other LUAD-specific ceRNA subnetworks

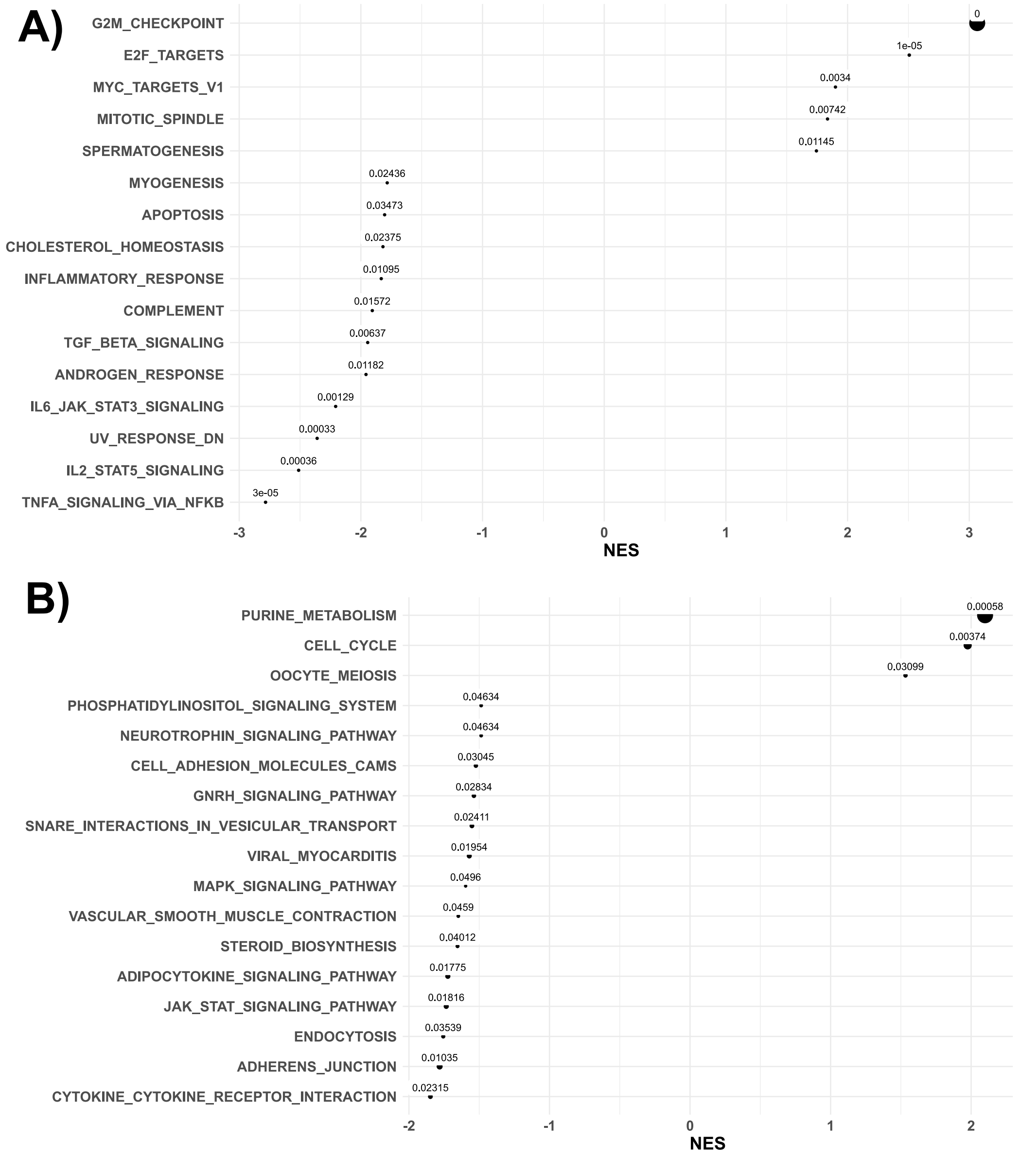

Figure S6: The (A) hallmark and (B) KEGG pathway enrichment results of LUAD-specific DeceRNAs

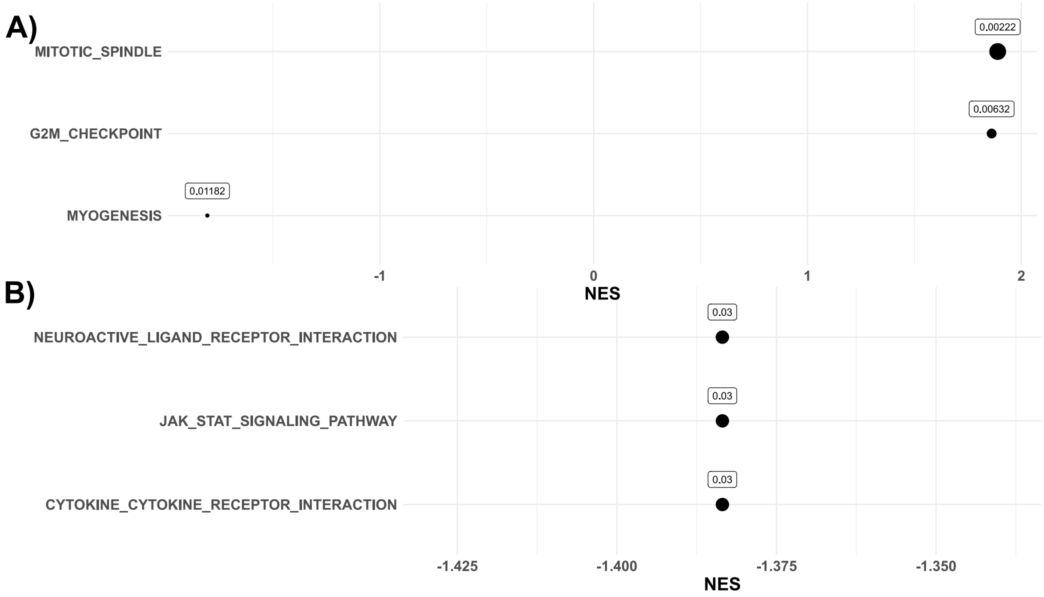

Figure S7: The (A) hallmark and (B) KEGG pathway enrichment results of BRCA-specific DeceRNAs

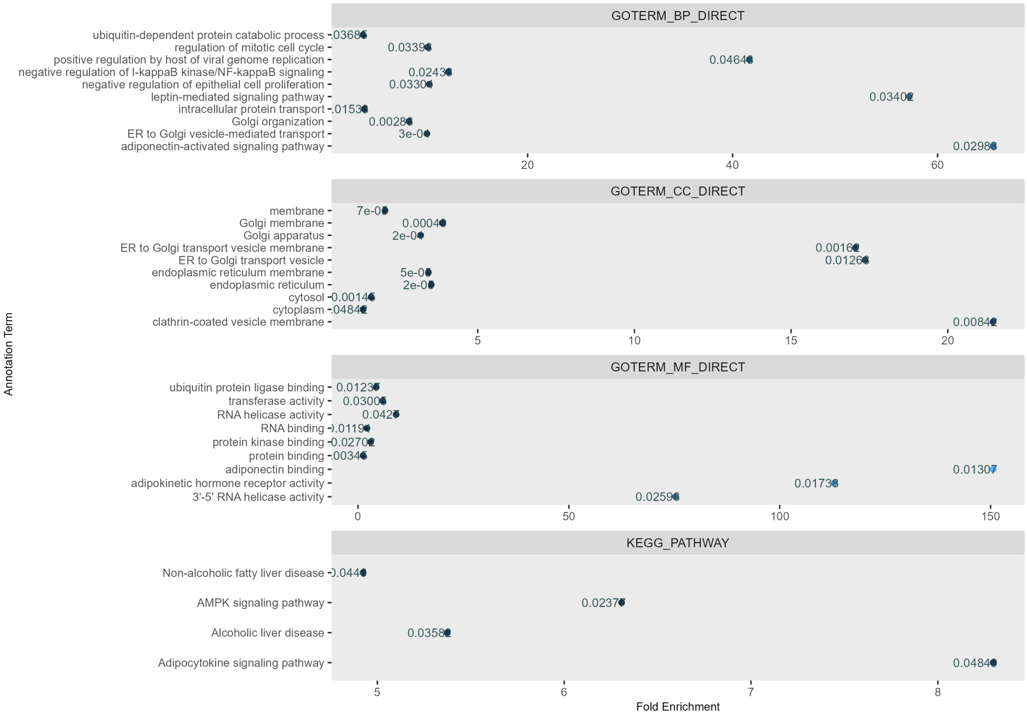

Figure S8: Functional annotation of common ceRNAs
